# Branched High Aspect Ratio Nanostructures Fabricated by Focused Helium Ion Beam Induced Deposition of an Insulator

**DOI:** 10.3390/mi12030232

**Published:** 2021-02-25

**Authors:** Frances I. Allen

**Affiliations:** 1 Department of Materials Science and Engineering, UC Berkeley, Berkeley, CA 94720, USA; francesallen@berkeley.edu; 2 California Institute for Quantitative Biosciences, UC Berkeley, Berkeley, CA 94720, USA; 3 National Center for Electron Microscopy, Molecular Foundry, Lawrence Berkeley National Laboratory, Berkeley, CA 94720, USA

**Keywords:** focused ion beam induced deposition, helium ion microscope, nanopillar

## Abstract

Helium ion beam induced deposition using the gaseous precursor pentamethylcyclopentasiloxane is employed to fabricate high aspect ratio insulator nanostructures (nanopillars and nanocylinders) that exhibit charge induced branching. The branched nanostructures are analyzed by transmission electron microscopy. It is found that the side branches form above a certain threshold height and that by increasing the flow rate of the precursor, the vertical growth rate and branching phenomenon can be significantly enhanced, with fractalesque branching patterns observed. The direct-write ion beam nanofabrication technique described herein offers a fast single-step method for the growth of high aspect ratio branched nanostructures with site-selective placement on the nanometer scale.

## 1. Introduction

Focused ion beams (FIB) are widely used for additive and subtractive nanofabrication, either via direct-write exposure, or using resist-based methods. In the direct-write approaches, the ion beam locally removes material by sputtering, or adds material by FIB-induced deposition (FIBID) [1]. In the latter, a gaseous precursor is injected into the microscope chamber and its molecules become dissociated upon interaction with the primary ions, scattered ions, and with the ion-induced secondary electrons [2]. The non-volatile reaction products form the deposits, and the volatile reaction products are pumped away. The majority of FIB work to date has been performed using gallium ions from a liquid-metal ion source, with much success, but recent introductions of FIB sources for other ion species are expanding the scope of FIB-based research at a rapid pace. New FIB sources include the plasma source (principally xenon ions) [3], liquid-metal alloy sources (gold, bismuth, silicon ions, etc.) [4], cold-atom-based sources (principally lithium and cesium ions) [5], and the gas field-ionization source (GFIS) (helium and neon ions) [6]. With the wide choice of ion species now available, along with a wide choice of other beam parameters such as beam energy and current, nanofabrication tasks can be optimized and new ones enabled by choosing the appropriate FIB.

For the deposition of electrically insulating structures, it is advantageous to use a non-metallic ion species, since ions are co-implanted with the deposited material and can thus affect the resistivity. Furthermore, if deposits with the smallest critical dimensions are required, then the GFIS-based instrument is an ideal choice, since it delivers a probe size down to 0.5 nm and produces minimized feature broadening due to the small interaction volume of the light ions near the sample surface [7]. To date, most FIBID of insulating materials using the helium/neon ions of the GFIS has focused on the deposition of broad-based pad structures to create electrically insulating barriers for circuit edit applications and device prototyping [8]. Early helium FIBID work also included exploratory studies of the deposition of insulator nanowires (grown along the surface of the substrate) [9] and nanopillars (grown vertically) [10]. In contrast, helium FIBID using metallic precursors is more advanced, incorporating both broad-based deposits [11,12,13], and a range of other structures including various nanowires [12,14,15,16,17], nanopillars [18,19,20,21,22,23,24], nanohelices [25], and nanoscale mesh-based polyhedra [26]. Moving forward, further work investigating helium FIBID of more complex insulator nanostructures is required.

In the early helium FIBID nanopillar studies mentioned above [10], it was found that nanopillars deposited from an insulator precursor exhibited irregular branch-like structures protruding from the main body, which is not observed for nanopillars deposited from a metallic precursor. This branching phenomenon was attributed to charging during the growth process, and similar effects have been observed for focused electron beam induced deposition (FEBID). For example, 15 years earlier, Banhart observed the growth of fractalesque carbon deposits in the transmission electron microscope (TEM) when focusing the electron beam onto the edge of insulating substrates [27,28]. A growth mechanism was proposed involving the aggregation of polarized and/or ionized precursor molecules along electric field gradients (the principal origin of the precursor molecules in the TEM case was concluded to be hydrocarbon contamination of the sample). Since then, and at around the same time as the first helium FIBID work, the fabrication of branched insulator nanopillars by FEBID in the scanning electron microscope (SEM) was also reported [29]. The authors found that charging and hence also branching of the nanopillars could be suppressed by co-injecting water into the microscope chamber together with the gaseous insulator precursor. Following this charge-neutralization approach, insulating nanopillars with smooth sidewalls were obtained. However, for future applications, branched nanopillars such as those created by FEBID or helium FIBID of an insulator may be of unique benefit, hence further investigation and tuning of this phenomenon is certainly warranted.

In this study, helium FIBID of high aspect ratio nanostructures (nanopillars and nanocylinders) using the insulator precursor pentamethylcyclopentasiloxane (PMCPS) is investigated, with particular focus on characterizing the resulting branched structures by TEM and investigating growth rate effects. The nanostructures are grown directly onto TEM-compatible substrates to allow direct imaging and elemental analysis at high spatial resolution without the need for further sample preparation steps. It is found that, by increasing the flow rate of the precursor, the vertical growth rate can be significantly enhanced and more complex branching morphologies are obtained.

## 2. Experimental Methods

The deposition experiments were performed using a Zeiss ORION NanoFab helium/neon/gallium FIB microscope (Carl Zeiss, Peabody, MA, USA), which incorporates a GFIS column for helium and neon FIB, and a conventional gallium FIB column. The GFIS was operated with a helium gas pressure of 1–5 × 10${}^{-6}$ Torr at 25 kV. The 10 µm beam-limiting aperture was selected to give a spot size on the sample of ∼0.5 nm with a beam current of 1–3 pA (spot control value set to 4). An OmniGIS II gas injector system from Oxford Instruments (Abingdon, UK) was used to inject the siloxane-based PMCPS gaseous precursor (cartridge at room temperature) towards the sample via a needle, the end of which was positioned approximately 100 µm from the target region on the sample. The chamber pressure during flow of the precursor gas was set to 2 × 10${}^{-6}$ Torr and 1 × 10${}^{-5}$ Torr in separate experiments. The former is the ‘standard’ chamber pressure targeted for this precursor and the latter is higher than is usually used.

NanoPatterning and Visualization Engine (NPVE) software from Fibics, Inc. (Ottawa, ON, Canada) was used to control the beam during the FIBID process, selecting ‘spot mode’ exposures to grow the nanopillars (continuous dwell, patterning doses of 10–80 µC/µm${}^{2}$) and annulus patterns to grow the nanocylinders (concentric mills with outer diameter 150 nm, ring thickness 1 nm, dwell time 1 µs, refresh time 10 µs (after each circular path), scan spacing 0.5 nm, with patterning doses of 10–80 nC/µm${}^{2}$). All structures were deposited sequentially. Note that the patterning doses quoted above are three orders of magnitude higher for the nanopillars than for the nanocylinders. This is because the ion dose values refer to the areal doses computed by the NPVE software based on the estimated beam diameter of 0.5 nm and the pattern size selected, i.e., these dose values do not correspond to the final cross-sectional area of the deposit, which is always larger due to ion beam scattering effects. A nanopillar deposited using a beam current of 1 pA and a patterning dose of 10 µC/µm${}^{2}$ took 2 s to complete, and a nanocylinder deposited using the same beam current and a patterning dose of 10 nC/µm${}^{2}$ took 7 s. For comparison, metallic nanopillars were also deposited, using the precursor tungsten hexacarbonyl, W(CO)${}_{6}$, (cartridge temperature 50 ${}^{\circ }$C) targeting a chamber pressure of 8 × 10${}^{-6}$ Torr (typical value for this precursor). Before each set of depositions, care was taken to achieve the best beam focus (smallest spot size) and to correct for any beam stigmation.

**Figure 1:**
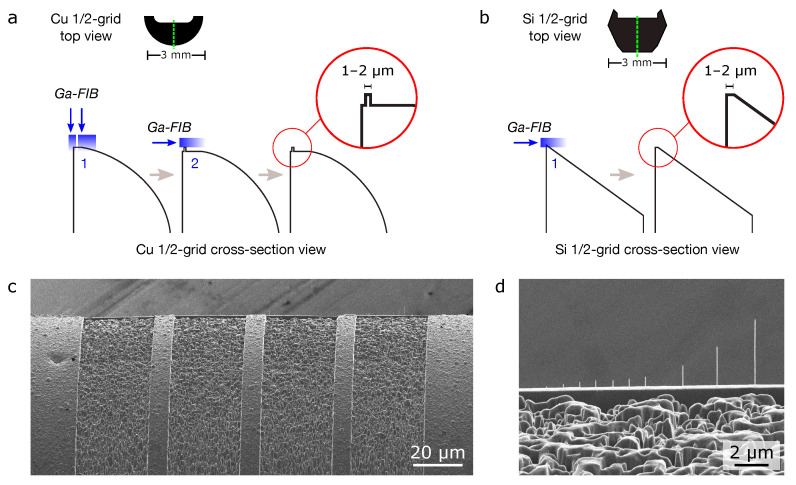
Preparation of TEM substrates for deposition experiments: (**a**,**b**) Schematics showing gallium FIB milling procedure to create flat and narrow platforms on the copper and silicon half-grids, respectively. The green dashed line in the top-down view of each grid marks the plane for the cross-section views (the latter are not drawn to scale). The first FIB milling step in (**a**) consisted of a coarse pre-mill at 1.5 nA followed by clean-up mills of the sides of the platform using beam currents down to 100 pA. In the second step, clean-up milling only of the top of the platform was performed at 100–300 pA. In (**b**), milling was performed at the lower currents only, since less material had to be removed. (**c**) Helium ion microscopy (HIM) view of four platforms milled into a copper grid following the method shown in (**a**). Each platform has a length of 30 µm and width of 1–2 µm. (**d**) Higher-magnification HIM view of nanopillars deposited onto the 4th platform in (**c**). In this example, the tallest nanopillar (metallic) was deposited using a patterning dose of 640 µC/µm${}^{2}$.

Two types of substrate were used for the experiments: 3 mm copper TEM slot-grids (PELCO, 0.4 × 2 mm slots, Ted Pella) and 3 mm silicon TEM half-grids (Dune Sciences). The copper grids were cut in two along the length of the slot, using a sharp razor blade to produce half-grids similar in shape to the silicon half-grids. The half-grids were mounted onto aluminum low-profile 36${}^{\circ }$ pin mounts (Ted Pella) using carbon tape. Flat and narrow platforms (width 1–2 µm) for the FIBID experiments were then machined into the grids using the gallium FIB of the ORION NanoFab microscope, as outlined in Figure 1, using appropriate stage tilts and rotations to achieve the milling directions required. For the deposition experiments, the stage was tilted such that the nanostructures were grown onto the flat FIB-milled platforms under normal incidence.

After the depositions, low-magnification images of the deposits were first acquired by helium ion microscopy (HIM; see e.g., Figure 1d). Then, each half-grid was mounted into a TEM sample holder using metal o-rings in a sandwich configuration. Bright-field TEM, dark-field scanning TEM (STEM), and selected-area electron diffraction were performed using an FEI TitanX electron microscope operated at 300 kV. Elemental mapping by X-ray energy-dispersive spectrometry (XEDS) was performed using the same microscope operated at 80 kV using an FEI Super-X quadrature X-ray detector and Bruker Esprit software.

## 3. Results and Discussion

### 3.1. Helium FIBID of High Aspect Ratio Insulator Nanostructures using the “Standard” Precursor Flow Rate

**Figure 2:**
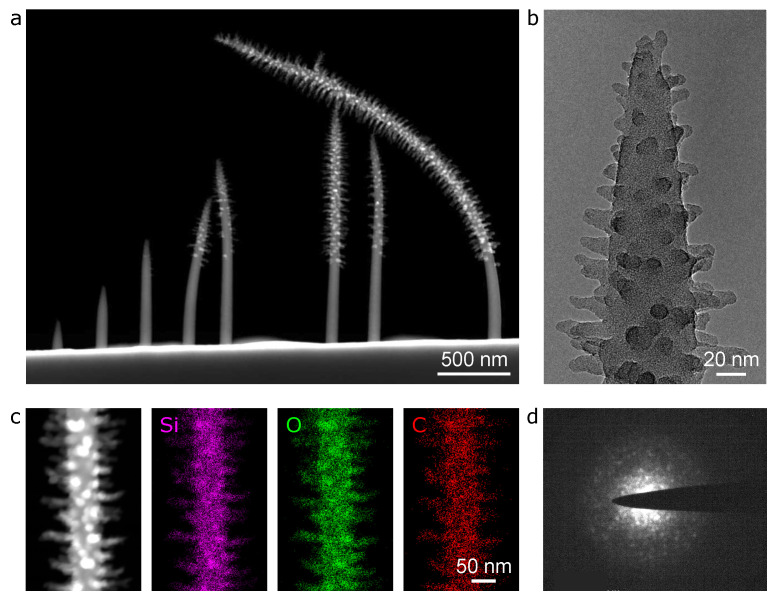
Insulator deposition of nanopillars by helium FIBID (chamber pressure 2 × 10${}^{-6}$ Torr, beam current 2.5 pA, copper substrate): (**a**) Dark-field STEM of a series of nanopillars (deposition times from left to right: 2, 4, 6, 8, 10, 12, 12 (repeat), and 16 s). (**b**) Bright-field TEM of the apex region of the 5th nanopillar in (**a**) (deposition time 10 s). (**c**) STEM-XEDS of branched trunk region of the 6th nanopillar in (**a**) (deposition time 12 s), showing the reference dark-field STEM image on the left and colorized maps for silicon, oxygen and carbon. (**d**) Selected area electron diffraction pattern for a single branch.

Figure 2 shows a set of (S)TEM images of helium FIBID PMCPS nanopillars grown using the lower of the two PMCPS flow rates tested (chamber pressure 2 × 10${}^{-6}$ Torr). The nanopillars increase in height with increasing dose, and above a certain threshold height, radial branches are formed. In the dark-field STEM image of Figure 2a, these branched structures are clearly evident for the fourth nanopillar from the left onwards. The threshold height for branch formation is ∼500 nm, implying that above this height, an electrostatic field of sufficient strength is established at the pillar apex to trigger the aggregation of polarized/ionized precursor molecules to form the branches [27]. Since the insulator deposits are continuously bombarded with positive ions and will subsequently emit secondary electrons, the charge accumulated will be positive. Tall pillars tended to bend, particularly during the imaging.

A higher magnification bright-field TEM view of the apex of a branched nanopillar is shown in Figure 2b and STEM-XEDS elemental mapping results for a branched region further down on the main body are shown in Figure 2c. The branch diameters are ∼10 nm and branch lengths on the tallest nanopillar reached 100 nm (shorter branches are observed near the apex, where the pillars start to taper). Since the precursor molecule, PMCPS, contains silicon, oxygen, and carbon, all of these elements are expected to be found in the deposits. For the region mapped in Figure 2c, the relative amounts of these elements are quantified as: Si 27.2 ± 0.5 at.%, O 42.9 ± 4.0 at.%, and C 29.9 ± 3.0 at.% (errors are 3-sigma values from the peak-fitting routine to the K-series). No difference in the composition of the branches versus that of the main body of the nanopillars was measured, nor any variation in composition versus pillar height. The base width of the PMCPS nanopillars is ∼80 nm. This is about twice the width generally reported for metallic nanopillars grown by helium FIBID using similar beam parameters [18,19,21,23,24], which may be related to the relatively high deposition efficiency of PMCPS [8].

In Figure 2d, the selected-area electron diffraction pattern for a single branch on a PMCPS nanopillar is shown. There are some random diffraction spots visible. This was not the case for the lower non-branched (trunk) regions of the nanopillars, which only showed amorphous rings in the diffraction patterns. The diffraction result in Figure 2d thus suggests that the branch structures comprise an amorphous matrix in which randomly oriented nanocrystals are also present.

**Figure 3:**
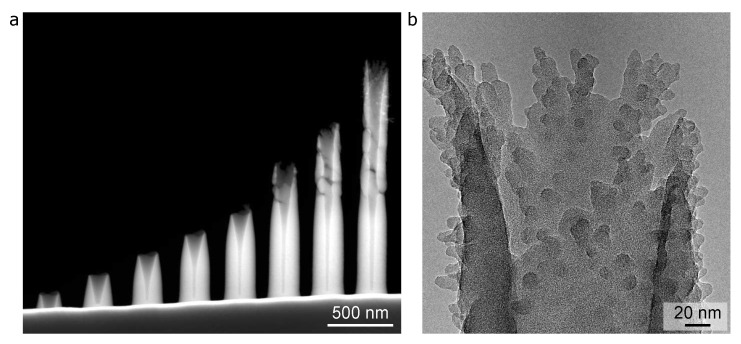
Insulator deposition of nanocylinders by helium FIBID (chamber pressure 2 × 10${}^{-6}$ Torr, beam current 2.5 pA, copper substrate): (**a**) Dark-field STEM of a series of nanocylinders (deposition times from left to right: 7, 14, 21, 28, 35, 42, 49, and 56 s). (**b**) Bright-field TEM of the apex region of the 8th nanocylinder in (**a**).

Figure 3 presents the results for PMCPS nanocylinders (as opposed to nanopillars) deposited using the same precursor flow rate as for Figure 2, and onto the same substrate, using the same beam current. In the dark-field STEM image of Figure 3a, it can be seen that as the height of the nanocylinders increases with dose, cracking starts to occur above a height of ∼700 nm (see the three pillars on the right). This transition coincides with an overall thinning of the nanocylinder sidewalls as they grow taller. A higher magnification bright-field TEM view of the tip region of the tallest nanocylinder is shown in Figure 3b. While branches are also observed, these form much higher than on the PMCPS nanopillars and tend to be shorter, indicating that, for the larger-volume nanocylinder deposits, the charging-induced branching effect is less pronounced. The outer diameter of the nanocylinders is ∼220 nm and the width of the sidewalls near the base is ∼80 nm, which equals the width of a single PMCPS nanopillar. This makes sense, because the annulus pattern used to grow the nanocylinders directed the beam onto a circular path that was only 1–2 pixels wide (a nanopillar is grown by deposition onto a single pixel, giving the same diameter).

### 3.2. Helium FIBID of High Aspect Ratio Insulator Nanostructures Using a Higher Precursor Flow Rate

**Figure 4:**
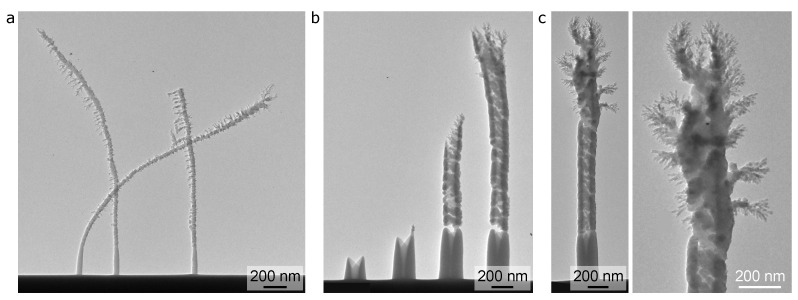
Insulator deposition of nanopillars and nanocylinders by helium FIBID using a higher precursor flow rate (chamber pressure 1 × 10${}^{-5}$ Torr, beam current 1 pA, silicon substrate): (**a**) Bright-field TEM of nanopillars (deposition times from left to right: 2, 2 (repeat), and 4 s—the latter snapped, hence the height is reduced). (**b**) Bright-field TEM of nanocylinders (deposition times from left to right: 7, 14, 21, and 28 s). (**c**) Bright-field TEM and higher-magnification view of an additional nanocylinder (deposition time 28 s).

An overview of the (S)TEM results for PMCPS nanopillars and nanocylinders grown using a higher precursor flow rate (raising the chamber pressure to 1 × 10${}^{-5}$ Torr) is shown in Figure 4. While these particular images are for depositions onto a silicon substrate using a slightly lower beam current of 1 pA, HIM imaging of a range of deposits grown onto each substrate for various beam currents has confirmed that the key factor causing the very different growth characteristics seen here (compared to Figure 2 and Figure 3) is the precursor flow rate. The PMCPS structures deposited with the higher precursor flow rate grew faster than those deposited using the lower precursor flow rate. For example, the two PMCPS nanopillars on the left of Figure 4a grown using a deposition time of 2 s reached ∼2400 nm in height, whereas the PMCPS nanopillar in Figure 2a grown for the same deposition time, but with the lower chamber pressure of 2 × 10${}^{-6}$ Torr, only reached a height of 215 nm. The tall PMCPS nanopillars seen in Figure 4a were also more prone to bending during imaging than their shorter counterparts, often leading to fracture. For example, the nanopillar on the right in Figure 4a was originally taller than the ones on the left, since it was grown using a higher beam dose, but it then fractured during imaging.

The threshold height for branching for the PMCPS nanopillars grown using the higher precursor flow rate is ∼300 nm and the branches themselves are much more random than those of the PMCPS nanopillars grown using the lower flow rate. While the branches still follow a radial growth pattern, there is preferential growth to one side (e.g., towards the left side of the image in Figure 4a), which could be due to the uni-directional flow of the gaseous precursor. Multibranched fractalesque structures also formed, especially in the upper regions. These results indicate that the charging-induced branching phenomenon is enhanced when the deposition rate is faster. Thus, precursor flow rate can be used to tune the overall vertical growth rate and also the extent of branching.

Considering the nanopillar widths, the base diameters of the helium FIBID PMCPS nanopillars that are grown with the higher precursor flow rate are ∼40 nm, i.e., half the diameter of the PMCPS nanopillars grown using the lower flow rate. This reduction in diameter can be attributed to the faster vertical growth rate, leaving less time for pillar broadening (the latter resulting from precursor dissociation induced by scattered ions and their associated secondary electrons as they exit the flanks of the pillar [18]).

The results for a series of PMCPS nanocylinders also grown using the higher precursor flow rate are presented in Figure 4b, where we see a bright-field TEM image of deposits for increasing dose from left to right. Note the nanocylinder second from the left possibly fractured upon imaging, although it is also possible that above this threshold height the growth rate increases, since then the sidewalls start to thin, i.e., the volume per unit length is reduced. The height of this nanocylinder (∼500 nm) represents the height above which severe cracking and narrowing of the cylinder sidewalls is observed. The taller nanocylinders appear to be only very weakly attached to their more robust bases at this threshold height. For these PMCPS nanocylinders grown using the higher precursor flow rate, the threshold height for thinning of the sidewalls and cracking was lower than for the nanocylinders that grew more slowly (Figure 3a). This also reflects the trend for the threshold height for branching on the nanopillars, which was also lower for the nanopillars deposited using the higher precursor flow rate. Thus, for both types of nanostructure, charging effects were enhanced when the growth rate increased. As for the outer diameters and sidewall base diameters of the nanocylinders, these are quite similar for the two flow rates tested. This is expected, because the enhancement in vertical growth rate for the nanocylinders is not as dramatic as it was for the nanopillars, hence sidewall broadening will not have been as significantly affected.

The PMCPS nanocylinder that is pictured in Figure 4c (bright-field TEM) was grown further apart from those in Figure 4b and will thus have been less affected by proximity effects from neighboring structures (which can also cause the nanostructures to grow towards one another). This nanocylinder exhibits the most complex fractalesque branching pattern observed in all the structures investigated, spanning the upper 1 µm-long segment of the deposit, as shown in more detail in the higher-magnification view.

Notably, in related FEBID experiments concentrating on PMCPS nanopillars fabricated using the electron beam of the SEM, de Boer et al. observed a large enhancement in branching when changing from a bulk silicon substrate (semiconducting) to a silicon nitride membrane (insulating) [29]. As mentioned above, no significant effect was observed in the present helium FIBID study when comparing copper and silicon substrates, although it would be interesting to test a highly resistive substrate like silicon nitride in the future. Compared with FEBID, the deposition efficiencies for helium FIBID are known to be at least an order of magnitude higher [19,30]. Hence, helium FIBID from an insulator precursor offers a faster method to fabricate branched high aspect ratio nanostructures than is possible using the FEBID-based method.

### 3.3. Comparison of Growth Rates for Helium FIBID Insulator Nanopillars and Nanocylinders

**Figure 5:**
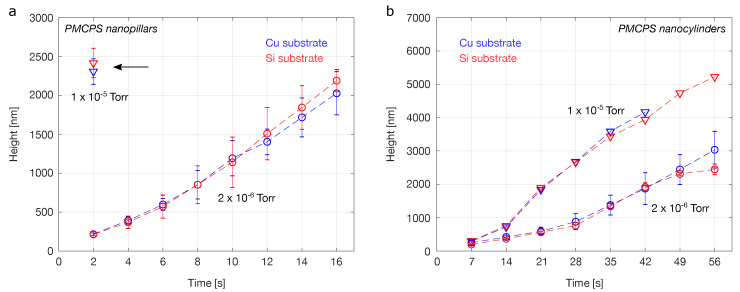
Plots of height versus deposition time for helium FIBID insulator nanostructures: (**a**) Nanopillars. (**b**) Nanocylinders. Data points for chamber pressures 2 × 10${}^{-6}$ Torr and 1 × 10${}^{-5}$ Torr are marked with circles and triangles, respectively. Blue represents data for the copper substrate and red shows data for the silicon substrate. The beam current was 1 pA throughout.

In order to investigate the growth rates in more detail, plots of nanostructure height versus deposition time are shown in Figure 5 for the two precursor flow rates investigated. These data points are for nanostructures grown using the same beam current (1 pA) throughout, since it is known that small changes in current in the picoamp range can influence the deposition rates of helium FIBID nanopillars [18,19] (albeit not as dramatically as the effect observed here for changing the precursor flow rate). The results for depositions on both the copper and the silicon substrates are shown. For data points corresponding to three or more duplicate runs, error bars corresponding to the standard deviations from the mean value are given.

For the PMCPS nanopillars grown using the ‘standard’ precursor flow rate (chamber pressure 2 × 10${}^{-6}$ Torr), a steady increase in pillar height with dose is observed (Figure 5a, circles). Within the experimental error, no discernible difference in growth rate is observed between the copper and silicon substrates. For the higher precursor flow rate (chamber pressure 1 × 10${}^{-5}$ Torr), the growth rate is much faster, as already indicated by the results shown previously in Figure 4. For these fast-growing nanopillars, a height is only plotted for growth using the lowest dose (deposition time 2 s), since these nanopillars tended to bend excessively and fracture. Again, no discernible difference in growth rate for the two substrates was observed. Compared with the nanopillars deposited using the lower precursor flow rate at the same dose, the nanopillars deposited using the higher precursor flow rate grew about 10× taller.

Deposition efficiencies and yields can also been computed to enable further comparison. In the case of the PMCPS nanopillars deposited using the lower of the two flow rates, a deposition efficiency approaching 0.09 nm${}^{3}$/ion is obtained. This is based on estimating the volume of the nanopillar, taking into account the taper at the apex. For the higher flow rate, a deposition efficiency of twice the previous value is estimated, of 0.18 nm${}^{3}$/ion. In terms of deposition yield, values of ∼0.56 and 1.07 µm${}^{3}$/nC are calculated for the lower and higher precursor flow rates, respectively. These values compare well with PMCPS deposition yields reported in the literature for helium FIBID of large-area deposits [8]. While these calculations show that the increase in precursor flow rate doubles the volumetric deposition yields, it is clear from Figure 5a that the effect on the vertical growth rate is much greater. This is because the volume scales with the radius squared; as determined from the TEM data, the radius of the faster-growing nanopillars is reduced by one half, meaning that doubling the volume actually increases the height by a factor of 8. The reason that we do not see a factor of 8 difference between the two sets of data for the deposition time of 2 s in Figure 5a (instead, the factor is closer to 10), is that the short nanopillar that is obtained at the lower precursor flow rate only comprises a tapered tip (smaller volume), since it has not yet grown tall enough for the cylindrical body to form (see Figure 2a).

In the case of the PMCPS nanocylinders (Figure 5b), those deposited using the higher precursor flow rate again grew taller, but the difference was less dramatic than for the nanopillars. Here, height measurements for a wider range of doses were possible, since overall the nanocylinders are more stable and not as prone to bending, etc. Finally, as was the case for the nanopillars, no discernible difference in growth rate was observed for the two substrates tested.

The deposition efficiencies for the nanocylinders for the lower and higher precursor flow rates are estimated at 0.21 and 0.38 nm${}^{3}$/ion, respectively. The corresponding deposition yields are 1.32 and 2.37 µm${}^{3}$/nC. These estimates take into account the evolving shapes of the nanocylinders (tapered tops and narrowing sidewalls). Compared with the nanopillars, the deposition efficiencies and yields are about a factor of 2 greater for the nanocylinder geometry. This can be attributed to the fact that as opposed to the continuous illumination used to deposit the nanopillars, the nanocylinders were deposited using a beam refresh time (i.e., pause) between the the circular scan paths, which enables replenishment of precursor molecules at the reaction site, thus mitigating precursor depletion effects [7,20].

### 3.4. Helium FIBID Nanopillars Deposited Using a Tungsten-Based Precursor

**Figure 6:**
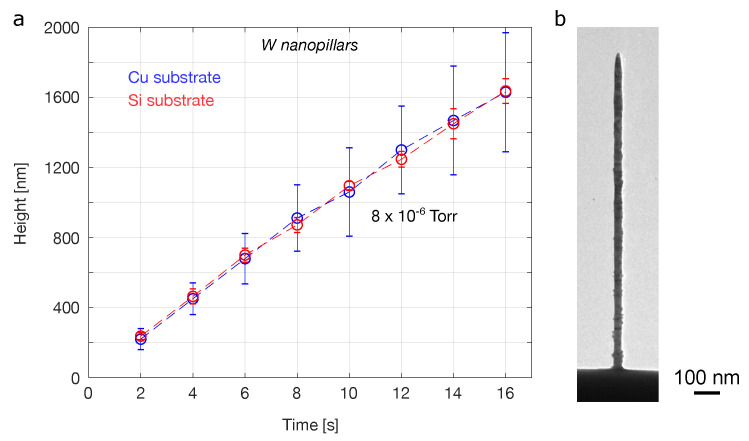
Deposition of metallic nanopillars from W(CO)${}_{6}$ precursor by helium FIBID: (**a**) Plot of height versus deposition time. Chamber pressure 8 × 10${}^{-6}$ Torr, beam current 1 pA. Blue and red data points represent values for the copper and silicon substrate, respectively. (**b**) Bright-field TEM image of a tungsten-based nanopillar deposited onto the silicon substrate using a deposition time of 16 s.

For reference, the deposition of metallic nanopillars using the precursor W(CO)${}_{6}$ was also investigated. Here, just one precursor flow rate was tested, corresponding to the usual settings implemented for this particular gas chemistry. A plot of nanopillar height versus deposition time for the tungsten-based nanopillars is shown in Figure 6a. Again, both copper and silicon substrates were tested, with no measurable effect on the growth rate discerned. The vertical growth rate of the tungsten nanopillars was similar to that of the insulator nanopillars grown using the lower of the two PMCPS flow rates (compare with Figure 5a). The deposition efficiency for the tungsten-based helium FIBID nanopillars investigated here is estimated at 0.02 nm${}^{3}$/ion, with a corresponding deposition yield of 0.14 µm${}^{3}$/nC (i.e., comparable to the values obtained for helium FIBID of platinum- and tungsten-based nanopillars elsewhere [18,19,21], but lower than the PMCPS deposition efficiencies/yields discussed above). Thus, in terms of volumetric growth rates, those for the PMCPS nanopillars were higher than for the tungsten-based ones.

A representative TEM image of a tungsten-based nanopillar deposited using the highest dose (deposition time 16 s) is shown in Figure 6b. As can be seen, the sidewalls of the metallic nanopillar do not exhibit the side-branching observed for the insulating nanopillars. However, the sidewalls of the metallic nanopillars are also not smooth, exhibiting small protrusions along their lengths up to about 10 nm in size. It has been proposed that sidewall roughness is due to secondary electron emission from the sides of the pillar during the growth process and possible aggregation of precursor molecules [31]. This is based on the observation that when depositing metallic nanopillars by gallium FIBID onto an insulating substrate (silicon nitride), sidewall roughness could be suppressed, attributed to suppressed secondary electron emission due to the positive charge accumulating on the surface of the electrically floating nanostructure. While no obvious difference in sidewall roughness was observed for the helium FIBID metallic nanopillars grown on copper versus silicon here, it is notable that the lower non-branched regions of the PMCPS nanopillars are actually a lot smoother than their metallic counterparts (see Figure 2a, at heights below ∼500 nm). This could also be a direct consequence of the charging effect, since the PMCPS nanopillars grown by helium FIBID will also become positively charged, thus similarly suppressing the emission of secondary electrons and the accompanying peripheral deposition. Above ∼500 nm, the mechanism of PMCPS precursor aggregation along electric field gradients then comes into effect.

The widths of the tungsten-based nanopillars were measured at ∼30–40 nm, irrespective of the substrate and in agreement with the widths of metallic (platinum and tungsten) helium FIBID nanopillars reported in the literature [18,19,21,23,24]. Beam focus is known to have an effect on nanopillar diameters, with tighter focus resulting in the narrowest pillars [20,21] and beam current can also be used to tune the nanopillar diameter [18,19,23,24]. However, the larger diameters of the PMCPS nanopillars seen in the present study (Figure 2) are primarily attributed to the precursor, as opposed to changes in beam focus or current. Finally, the PMCPS nanopillars deposited using the lower precursor flow rate have longer tapered end-segments than the tungsten-based ones.

## 4. Summary and Conclusions

This study has shown that insulating high aspect ratio nanostructures grown by helium FIBID exhibit a branching phenomenon attributed to charging that is not observed for their metallic counterparts. Branching occurs above a certain threshold height and branching behavior can be significantly enhanced by increasing the flow rate of the gaseous precursor, leading to faster growth and the formation of complex fractal-like branching patterns. Diffraction analysis of the branch structures reveals a nanocrystalline component in the otherwise amorphous material. For applications requiring high aspect ratio nanopillars with smooth sidewalls, charge-neutralization strategies may be applied. However, other applications may directly benefit from branched nanopillars such as those demonstrated here, which can be deposited efficiently at precisely defined locations in a single-step process using the helium FIBID method.
